# "The Hospital" Nursing Mirror

**Published:** 1899-02-25

**Authors:** 


"The Hospital" Nursing Mirror.

Being the Nursing Section of "The Hospital."

[Contributions for this Section of "The Hospital" should be addressed to the Editor, The Hospital, 28 & 29, Southampton Street, Strand, London, W.C., and should have the word "Nursing" plainly written in left-hand top corner of the envelope.]

### News from the Nursing World.

### GUY'S HOSPITAL ANNUAL CONCERT.

The Nurses' Choral Society and the Students' Glee Club gave their third annual concert last week, on two successive evenings, and must be congratulated on a most excellent entertainment. Considering how little time is available for busy hospital workers, the Guy's nurses and students deserve all praise for their rendering of Macfarren's cantata "May Day," which was the first part of their programme, and the part songs and quartettes which followed. Sister Christine's and Nurse Daniels' solos and songs met with great applause, as did those of Mr. H. J. Starling and Mr. H. McD. Parrott. The Governor's Court Room, where the concerts were held, was well filled both nights. The proceeds, after defraying the season's expenses, will go to the nurses' piano fund.

### ARMY NURSING NEWS.

Two new spheres of work have lately been thrown open to the Army Nursing Sisters. A few months ago a staff, consisting of Sister Russell (superintendent) and Sisters Potts and Marton, were sent on duty at the station hospital, Colchester; and last month Superintendent Sister Dowse, accompanied by Sisters Noble and Bond, were ordered to South Africa; they will be stationed at Natal. Now that the Soudan campaign is over the staff at the citadel hospital, Cairo, has been reduced, and Sister Harper, who was sent home just before Christmas in charge of some of the wounded, will not return there. She will be sent out instead to fill the vacancy at Malta caused by the resignation of Sister Bloomer. Sister Harper has been working for two years at Cairo, and has therefore to serve three years more before her five years' term of foreign duty is accomplished.

### YORK HOME FOR NURSES.

The Dean of York presided over the annual meeting of the York Home for Nurses which was held at the Institution, 37, Monkgate, on the 13th inst. There were also present: Rev. Canon Watson, the Lady Mayoress, Lady Emma Purey-Cust, and other influential residents in the neighbourhood. The report showed that the staff consisted of a sister superior, an assistant sister, 62 nurses, five district nurses, and 22 probationers; also that the work had been fully and efficiently maintained during the year. The nurses now wear the badge of the "White Rose of York" below the left shoulder. Last autumn the debt of £200 on the home was discharged, and in spite of this heavy outlay there is a balance in hand of £28. The house, which was an expensive one, has been bought during the last thirteen years, and the payment in September was the last instalment. The York Home for Nurses is 30 years old, and enjoys a considerable measure of public sympathy and support. The greatest care is taken in the choice of probationers, who are trained in the best hospitals. The medical officer drew attention to the facts that 70 cases of consumption had been attended during the year; that five of their nurses had been employed at the Scarborough Sanatorium; that others of the staff had had charge of the South Bank Smallpox Hospital during the epidemic of smallpox in the winter and spring; whilst the York County Hospital had been supplied by their institution with nurses for some months. There is also a home hospital for paying patients in connection with the home. The institution is managed by the Sisterhood of the Holy Cross. Mr. W. Draper, in seconding the vote of thanks to the chairman, proposed that male nurses should be added to the staff. "Would ladies like to be nursed by men?" he asked; "then, if not, why should sensitive, delicately-minded elderly gentlemen be handed over to the absolute care of a female nurse of 25 or 27? Would not a nervous and bashful youth have the distress of his illness increased by the necessary offices of a watchful female attendant?"

### THE WORKHOUSE MATRON.

A lively correspondence is being carried on in the columns of the *Councillor and Guardian* on the vexed topic of the "Workhouse Matron," in which Miss Louisa Twining takes part. She deprecates the attempt to put "new wine into old bottles," declaring that the rules framed twenty years ago are inadequate to meet the new conditions created by the new order issued less than two years ago. She also draws attention to the irrelevance of judging trained nurses generally by individuals, as if anyone supposed that nurses could be perfect any more than other classes, especially when entering upon new and untried conditions of work. "These conditions must be more clearly defined if harmony instead of friction is to prevail, and considering what human nature is, we cannot be surprised if the latter spirit is too often now prevalent." She commends the idea of removing the sick away from the workhouse, and leaving them entirely in the nurses' care, and reverts to her old suggestion that workhouse infirmaries should be established as convenient central institutions into which the sick of the district could be gathered—a principle long since carried out with regard to lunatics, partial imbeciles, and schools. This would, in her opinion, remove the graver difficulties in the nurses' way, and would be likely to secure a good class of probationers. She again urges the boards of guardians to combine either in connection with the Poor Law Officers' Association or with the Poor Law Unions Association, and force some measures of reform upon the Central Board, and in such an event she says that some members of a voluntary association are willing to help with their experience of twenty years. This is another straw which shows that some members of the Workhouse Infirmary Nursing Association are keeping vigilant watch over all affairs relating to workhouse nurses, and are prepared to take action at the first favourable opportunity.

### RECOGNISED TRAINING.

The following questions, which have lately been asked of the Local Government Board, and the official answer which has been received, will be read with interest by all nurses who are proposing to seek work under the Poor Law. The questions which were put by our correspondent were as follow: "(1) Where can I obtain a list of the Training Schools for Nurses, recognised by the Local Government Board as qualifying nurses trained in them for the position of superintendent nurse? (2) Is the Local Government Board prepared to recognise certificates from more than one school, but showing that the combined training has occupied three years at approved schools as sufficient qualification for the post of superintendent nurse?" Below is the answer received:—

"February 21st, 1899.

"Madam,—In reply to yours of the 18th inst., I am directed by Dr. Downes to inform you that—(1) So far as he is aware no list of training schools has been issued by the Local Government Board, but that nearly all the Poor-Law infirmaries of the metropolis have already made, or are making, arrangements for training probationers; (2) that probably sufficient certificates of training, properly combined in more than one institution, would be valid under Article III. of the Order of August 6th, 1897.—I am, madam, yours obediently, "Harry W. Todd,
"Clerk to Dr. Downes, Inspector."

### ONE RESULT OF CHRISTIAN SCIENCE.

"Truth" publishes an appalling story. A gentleman met with an accident which resulted in his being left with one leg shorter than the other. Medical science failed to put him to rights, and in despair he determined to try what Christian science could do. He was introduced to a "healer," who was said to have had miraculous successes with similar cases. Unfortunately the lady had engagements on the Continent, and was only able to see him personally once. She, however, commenced the treatment at this interview, and departed for the Continent, promising to continue the course *in absentiâ*. The attentive reader of *Truth* is aware that in Christian Science absent treatment is very much the same as present treatment. So it proved in this instance. The leg began to grow. It continued to grow. It got as long as the other, but it showed no disposition to stop growing at that point. The owner became alarmed. He made inquiries after the absent healer, but failed to find her. His leg kept on growing, and in despair he advertised in the newspapers in the hope of stopping the absent treatment, but without success. His leg is now three inches longer than the other, and is still growing.

### TRAINING PROBATIONERS.

An instructive point in the training of probationers was discussed by the Cape Medical Council some little time ago. A medical man founded what would be called in England a hydropathic establishment, and pleaded urgently with the Council that they should recognise it as a "training school for nurses." It was argued that the young women received regular instruction, and obtained a great deal of experience in the course of five years' service; but what was urged on the other side was overwhelmingly against the proposition. What comparison could there be between the experience gained in an institution where convalescents congregate to grow well and amuse themselves and in a general hospital where each vacant bed is immediately filled with a *bonâ fide* patient? How could the instruction be as advanced and complete in a home where a case is the exception and not the rule, and where one medical man could easily overlook the whole? "We might as well," truly said the President of the Council, "licence the ships that carry doctors as training schools!" Candidates for training are often tempted in England to enter various nursing homes and cottage hospitals which cannot train nurses efficiently because they are not qualified to do so. These institutions take the trouble to teach ignorant, and therefore troublesome, probationers because trained nurses are expensive. Those who accept such training, however, cannot thus really qualify themselves as nurses. It is no use blinking at facts. Let them be warned in time. The unqualified nurse has a hard battle; she it is who swells the ranks of the unemployed, and finds the door to every promotion shut in her face.

### SHORT ITEMS.

An entertainment was given to the in-patients of the Cancer Hospital, Fulham Road, last Thursday evening by Mrs. Arthur Read. It consisted of the play "Liberty Hall," which was well acted under the direction of Mr. T. J. Lintott. During the past ten years Mrs. Read has often given the in-patients similar treats, which are always much appreciated.—An anonymous bequest of £100 made to the Guildford Nursing Institution is to be invested as a special emergency reserve fund.—Some re-arrangements of the staff at Caistor Workhouse have been made. The nurse has had her salary raised to £35, and an assistant nurse has been appointed. The number of inmates is decreasing.—Dr. O'Callaghan, the Roman Catholic Bishop of Cork, has invited the community of the Little Sisters of the Assumption to open a home for district nursing amongst the poorest inhabitants of Cork.—The Peterborough Nursing Association is richer to the extent of £4 10s. by Madame Stirling's recent concert given at that city.—A variety of sources have contributed to swell the funds of the Camborne Nursing Association Recently a jumble sale, followed by an entertainment, in the Assembly Rooms produced £11, and the gate-money at an approaching football match will be devoted to the same purpose.—On Tuesday, February 14th, Miss Hallowes, Hon. Treasurer of the Church Nurses' Guild, kindly invited those members of the guild residing within the London district to spend the evening at her house. Music and singing were enjoyed, and a helpful address was given by the Rev. E. Grose Hodge, Rector of Holy Trinity, St. Marylebone, who has lately joined the Council of the Guild.—Lady Raglan is interesting herself in St. Mary's Children's Hospital, Plaistow, E. She is getting up a bazaar at the Empress Rooms, Kensington, next month to raise money for it.—Number 6 of the course of Winter Entertainments at the National Hospital for the Paralysed and Epileptic took place on the 23rd inst., when an attractive programme of music and songs was given.

### Hints on the Home Nursing of Sick Children.

By J. D. E. Mortimer, M.B., F.R.C.S., formerly Surgical Registrar, &c., at the Hospital for Sick Children, Great Ormond Street.

(*Continued from page* 213.)

### VARICELLA (CHICKEN-POX)—ACUTE RHEUMATISM—RHEUMATIC FEVER—HEART DISEASE—BRONCHITIS, PNEUMONIA.

### Enteric (Typhoid) Fever—*continued*.

Too much starchy food is apt to produce flatulence. The constipation which is now likely to result from the weakened state may cause a high temperature and make the child very ill if neglected; but the nurse should never give an aperient on her own responsibility. I need not dwell on the serious or even fatal results of giving strong purgatives, or food unsuitable, either in quality or quantity, during convalescence. After a severe attack the mind is often considerably weakened, but this will, as a rule, gradually improve as the general strength is regained; and until this is established it is better not to take special measures to try and restore it.

### Varicella (Chicken-pox).

This is usually a very mild affair, but some means must be adopted to prevent scratching of the face, otherwise ugly little scars may be left. The same precaution is needed in variola (small-pox).

### Acute Rheumatism.—Rheumatic Fever.

In children, sweating, pain and swelling of joints are seldom marked, as in adults; the complaint shows itself more in other ways, such as tonsilitis, erythematous or other rashes, and more particularly by attacking the heart. The child must be kept lying in flannel between blankets and have a milk and farinaceous diet—no soups, beef tea, meat essence, tea, coffee, &c. Affected joints should be wrapped in cotton-wool as long as there is even a suspicion of rheumatism, and for some days after symptoms have subsided this treatment must be insisted on, although it may seem over-cautious, on account of the liability to peri-endocarditis. For the same reason any pain or uneasiness about the chest, dry cough, or hurried breathing should be at once reported to the doctor. Later on, any stiffness may be dealt with by fomentations, rubbing, and kneading. Cold, damp, and over-fatigue must be avoided. Warm underclothing is required, and the amount of meat in the diet should be limited.

### Heart Disease (Pericarditis and Endocarditis).

In the acute stages, and for some time afterwards, rest in the widest sense of the term is imperatively required. "The visit of a friend, some addition to the diet, neglect of the bowels, getting out of bed, even the fatigue of holding up a book to read, may be followed by some serious relapse or complication." Even with the most scrupulous care these are prone to occur, and the patience of nurse and relatives may be considerably tried. Nourishment must be fluid, given in smaller quantities and at shorter intervals than in most diseases. Attention must be paid to the proper warmth of the room (as near 60 deg. as possible). The bowels must be moderately relaxed to avoid straining. The nurse should be keenly on the watch for any increase of uneasiness as expressed in the face or attitude, and for any alteration in the colour of the lips or warmth of the extremities, or any working of the nostrils or other signs of difficult breathing. The child usually needs to be well propped up in bed with the head thrown rather back.

When recovery is taking place there must still be avoidance of over-exertion and excitement and precautions against cold. Much harm is often done by children being allowed to overtire and overheat themselves when first allowed to go out, especially at the seaside. The food must still be selected—bulky, innutritious articles being avoided—and drink taken preferably rather at a short interval before a meal than with it, so that the stomach is not over-distended. In this stage, and in chronic cases, there is apt to be over-feeding from a mistaken idea of keeping up the strength, and much firmness may be needed to prevent such. There must be no tight clothing.

After an early light breakfast, say of a rusk and a little warm milk, it is a good plan to rub systematically with a warmed Turkish towel, beginning with the feet and working upwards. The little patient, if old enough, may take the ends of the towel, folded lengthwise, and work at the back and neck, with the arms up (but there must be no stooping), then, after a sponge down with warm water, there should be rest on the bed or couch. Massage may be ordered, or various movements—passive, active, or resisted. These must be carried out in a moderately-warmed, well-ventilated room, and it is of great consequence that the patient take full, deep breaths all through; and they should not be continued if there is any drawing of lips or working of nostrils, any duskiness or pallor of face, palpitation, or other signs of distress, however slight.

### Bronchitis, Pneumonia.

The general management is the same as in adults. Care must be taken that the respiratory movements are not impeded by heavy covering (shawls, counterpanes) or a tight binder. Linseed poultices, especially jacket poultices, are now used much less than formerly. Their value is doubtful, and, besides being troublesome to both patient and nurse, the movements of the chest wall may be seriously hampered, especially in weakly babies, by their weight and by the swathing which is necessary to keep them in place. If a warm application is needed fomentations are preferable. The nurse may have to rub the chest with liniment, and in doing so it is a good plan to make intermittent pressure on the bases of the lungs by drawing the hand rather firmly from spine to sternum over the lower ribs. If a wool jacket is required cotton-wool (a cheap kind will do) may be quilted into the inside of an under-garment, or a piece of soft muslin should be taken large enough to cover the whole thorax. After this has been shaped to fit round the neck a sheet of cotton-wool should be laid on it and the edges turned over and tacked all round, also at the armholes. For examination of the lungs it is generally better that this should open at the back. In case of pneumonia, sponging or packing may be needed on account of high temperature, or the doctor may order an icebag to be applied to the affected part of the chest; if so, the nurse should ask him to what degree the temperature should be allowed to drop before removing it. The bag should be kept in place by a flannel binder. She should take the temperature frequently, say, every half-hour for the first two hours, then hourly, and should notice if the pain, &c., seems to be relieved. The feet and abdomen must be kept warm with hot water bottles, and if there is any blueness or shivering the bag must be taken away. If the time of the doctor's visit is known beforehand the child should be got ready for examination by having the clothes loosened—in case of a baby it is better to remove them altogether and for the nurse to have the small patient wrapped in a blanket on her lap by the fire; the back can be best examined when the baby's arms are round the nurse's neck with its head on her shoulder.

(*To be continued.*)

### Home Nursing Lectures in a Prison.

By Mrs. Will C. Hawksley.

A most interesting experiment is that which has of late been carried out by Miss Honnor Morten at Wormwood Scrubs Prison on behalf of the female criminals there located. So far as adult offenders are concerned, it may, indeed, be regarded as amongst the earliest active attempts, apart from those connected with religious influences, to render the period of punishment a reformative as distinct from a merely retributive epoch in the lives of the prisoners.

The last of a series of six lectures—the second series which has been given—upon simple subjects connected with domestic nursing and sanitation has recently been delivered in the otherwise unoccupied isolation hospital of the prison. The audience who listened a few months since to the earlier instructions was composed of somewhat hardened, and as a rule elderly, offenders against law and order. The effect produced, however, even upon that decidedly unpromising band, was so satisfactory as to induce the powers that be to invite the lecturer to repeat her efforts in another quarter. This suggestion, however, Miss Morten, influenced by the conscientious consideration that every labourer is worthy of hire, refused to accept unless, in return, an adequate payment could be made. She had been originally induced to move in the matter in consequence of certain recommendations contained in the then quite newly published report of the Special Prison Commission, and had gladly tendered the services which rendered possible a preliminary trial of the scheme. A second step should, she believed, however, tend to pave the way to a permanent and larger design.

Thereupon it became evident that there were at the disposal of the Prison Commissioners absolutely no funds which could be devoted to this good purpose. It was at once determined that the matter should, at the earliest possible moment, be brought to the attention of Parliament, with a view to the inclusion of some provision to meet necessary expenses in future Estimates. Meanwhile, however, the National Health Society, rather than suffer the present opportunity to be lost, consented to supply the lacking wherewithal. With the consequence that a second course, also at Wormwood Scrubs, was begun, and has now been brought to a conclusion. Moreover, owing to the fact that those under tuition have been selected from amongst the ranks of the First Offenders, and have therefore proved, as a rule, considerably younger and more teachable than their predecessors, greater results may reasonably be expected eventually to ensue from the latter than the former endeavour.

The curriculum has of necessity been elementary. It has included instruction as to (1) a healthy room—fresh air; (2) the patient—a day's round; (3) fevers—how we "catch" an illness; (4) accidents; (5) babies; (6) food. At the same time, though without any verbal application or mention of a moral, indirect lessons have been inculcated touching (1) good temper and cheerfulness; (2) regularity and order; (3) duty to neighbours; (4) self-control; (5) parental responsibility; (6) temperance. How far such good teaching may affect the future life of the pupils must, perforce, remain for the outer world to discover and to prove. That it has for the moment, however, been received with attention and even with eagerness was most obvious to myself when I, by grace of the Prison Commissioners, was upon one occasion admitted to the lecture-room to listen and to observe. Thus far I have been, so far as I am aware, the sole spectator thus privileged.

The scene was curious and unique. Wearing their prison uniform, with the red star of the First Offender (which is therefore also the Star of Hope) upon the front of each white cap and upon each left arm, the women were ranged in rows facing the lecturer. Female warders, in a proportion of about one to each four of their charges, stood or sat about the room, giving as keen heed to the information imparted as did those for whose special advantage the function had been organised. Whilst bandages, the material for poultices, and divers other paraphernalia were scattered upon a table well in view.

The proceedings began by a string of questions on the part of Miss Morten upon the subject of preceding addresses. The lecture itself immediately followed, though the questions even here did not entirely cease. Answers were marked by genuine intelligence and grasp of the subject. Thus, for instance, the woman who replied to an inquiry relating to the disadvantage, of brandy when used internally as a cure for a gaping and bleeding wound by the assertion that alcohol "makes the heart go faster," evinced a real insight into cause and effect that spoke well for both teacher and taught. Finally, manual dexterity was promoted by the spreading of linseed and mustard poultices, the application of head, thumb, and ankle bandages; in short, by the reduction of theory to practice. "You like the lectures?" I asked one of the scholars, a young woman who had followed every word and movement with avidity. "Oh, yes," she said, brightly. And, indeed, there was sufficient evidence in the countenances around, not only of a passing pleasure in the change thus brought into the monotony of prison life, but also, surely, of the softening and humanising result of even a small amount of education and of training. It was a pleasant-faced, quiet-mannered little company that, at the conclusion of affairs, marched out of the room, each member thereof bearing in her arms the well-scrubbed stool upon which she had been seated during the lecture. Can it be doubted that the work thus auspiciously begun is worthy of all encouragement and extension?

Of Miss Honnor Morten herself, who has been the actual originator of the effort, it is not needful to write at any great length. Her life has been as varied in its interests as useful in its outcome. Herself partly trained as a nurse, she was for some years on the staff of The Hospital, subsequently to which she became honorary secretary of the Nurses' Co-operative movement, and founded the Association of Asylum Workers. She was for three years a health lecturer under the London County Council, and is the author of several books and pamphlets, including "How to Become a Nurse" and "The Nurses' Dictionary." She is at present a member of the London School Board, having been returned at the head of the poll for the Hackney Division at the time of her earliest candidature in 1895. At that time, and just before the election, it was that she wrote expressing her intention "if I get on to make the health conditions of schools my chief points." It is an intention which, as all those who have of recent years watched with interest the discussions of the Board are aware, has been carried into good effect.

### Our Convalescent Fund.

We acknowledge with many thanks contributions to our Convalescent Fund of 5s. from Nurse Harvey; and of 5s. from Policy No. 4,598. We have just had the pleasure of hearing that our last convalescent, although not yet very strong, has been able to undertake work again, through the help the rest has given her. Our contributors will be glad to know this.

### Central Poor Law Conference.

### THE NURSING QUESTION.

Many interesting questions connected with Poor Law administration were discussed at the Central Conference of Guardians, held in the Council Chamber of the Guildhall on Thursday and Friday in last week, when Lord Beauchamp, who was in the chair, gave voice to the opinion of most enlightened people when he expressed his conviction that a new set of social circumstances had made the Poor Law somewhat out of date, and resulted in an impossible attempt to treat two classes, the deserving and the undeserving poor, under one rule. But to readers of The Hospital the chief interest of the conference will centre in the discussion upon "The Best Means of Providing and Training Nurses for the Indoor Poor," which occupied the afternoon of the first day.

A paper by Miss Wilkie, lady superintendent of the Halifax Workhouse Infirmary, dealt with the question of the moment—the present inadequate supply of trained nurses qualified to fulfil the requirements of the Nursing Order of 1897. Miss Wilkie deplored the lack of some uniform standard of training and attainment, and urged the constitution by the Local Government Board of a Nursing Department, worked by a committee of professional and lay members, by whom a general scheme of training might be formulated, and which should be a central examining body. She suggested that the age for receiving probationers should be decided by the department; the time of training to be four years, during the first two of which no salary should be paid; board, lodging, washing, and uniform provided, and training fees charged. There should be a staff of nursing inspectors to visit the infirmaries and examine the nurses in practical work, examinations to be on regular and fixed lines and at stated intervals, while certificates should be granted at the end of two years. Miss Wilkie thought the difficulty of finding nurses for small rural infirmaries would be overcome by grouping the infirmaries in districts, each possessing a central training school, from which nurses would be sent to the smaller workhouses in turn as they go from ward to ward in the school. The latter part of the paper dealt with the difficulty of the untrained matron, and pointed out the injustice of demanding evidence of definite training from nurses and then placing them under untrained authority. Miss Wilkie suggested that the Local Government Board should make it compulsory that the matron of a workhouse having an insufficient number of sick to warrant the employment of a superintendent nurse should be herself a trained and certificated nurse, besides having received special training in the duties of a matron, and that in the small workhouse the two offices might be combined.

Dr. Blake Maurice, guardian and late medical officer of the Marlborough Union, followed with a paper on the same subject, dealing with it, however, hardly from a practical standpoint. An interesting discussion followed, from which it was certainly encouraging to gather that a fairly general opinion prevailed that no inconsiderable part of the present difficulty in obtaining trained nurses for rural workhouses would be dissipated by a little trouble on the part of the Guardians. One speaker after another expressed the conviction that if the nurses were better paid, better fed, and better housed many more would be available, even under present conditions.

Lady Baker thought that the "second grade" nurse was the solution of the problem, alleging that it was a "shame" to expect highly trained nurses to "potter after poor old paupers" (!) and she urged the advantages of obtaining scholarships in nursing through the County Council Technical Committee. Several speakers dissented strongly from Miss Wilkie's suggestion of payment for training, and Sir Edmund Verney expressed his belief, with which all trained nurses will concur, that there can be no such thing as a "second grade" "nurse." The Rev. Henry Taylor was doubtful of the wisdom of setting up a huge official system by the proposed establishment of a nursing department, believing that too much officialism was an evil to be avoided. Miss Baker, of the Holborn Union, emphatically objected to the inferior class of nurse, and pointed out that the present undefined position of the trained nurse under the authority of the untrained matron was really the crux of the whole difficulty.

Miss Catherine Wood said that the difficulty of obtaining nurses applied chiefly to rural workhouses, and this would continue until the large infirmaries were compelled to train for the smaller ones. She thought the Local Government Board might be asked to schedule recognised training schools. It had been said that nurses would not face the monotony and isolation of workhouse life in rural districts, a statement which she emphatically contradicted; nurses were ready and anxious to enter the service of the Poor Law if once their position were properly defined and impossible conditions removed. Miss Wood condemned the plan followed by some Boards of Guardians of taking the selection of the probationers into their own hands, and pointed out that competent and suitable women would not apply if they knew their selection rested with untrained committees.

### Minor Appointments.

Bath Statutory Hospital for Infectious Diseases.—Miss Josephine Whittaker has been appointed Head Nurse of this institution. She was trained at the North-Eastern Hospital, London, at the London Fever Hospital, and at the Plymouth General Hospital. She held appointments at the London Fever Hospital for five years, and at the Nottingham Isolation Hospital for one and a half years.

Cumberland Infirmary, Carlisle.—Miss Christina A. M. Young was appointed Sister of the male wards at the above infirmary on the 14th inst. She was trained at the Bradford Royal Infirmary, and has since held the post of charge nurse of the male wards and theatre at the Rochdale Infirmary, Lancashire.

Buckhurst Hill Surgical and Medical Home.—Miss S. J. Deekes was appointed Nurse-Matron here on the 14th inst. She was trained at the Seaman's Hospital, Greenwich. She has been charge nurse at King's Lynn Hospital, and head nurse at Scarborough Hospital and Dispensary.

Isolation Hospital, Blaby, Leicester.—On January 18th Miss E. V. Godfrey was appointed Charge Nurse of the above. She was trained at Monsall and Lowestoft Fever Hospitals, and has been charge nurse at Leyton Hospital, and at Worcester City Hospital.

Fulham Union Infirmary, Hammersmith.—On the 16th inst. Miss Mary Ann Tarling was appointed Sister here. She was trained at Birmingham Workhouse Infirmary. She has been staff nurse at the Brentford Infirmary, Isleworth, and charge nurse at the Portsea Island Infirmary.

South Shields Union Infirmary.—Miss Mary Castell was appointed Charge Nurse here on February 16th. She was trained and has since held the position of staff nurse at the Mile End Infirmary, London, E.

### How Not to Obtain Poor Law Nurses.

EVENTS are pointing towards a crisis in the Poor Law nursing difficulty, a crisis long expected by the initiated, the arrival of which has only been a question of time. In the rural districts there can be no hope of any real solution of the problem until the present system of placing trained nurses under untrained matrons has been discarded in favour of one more in accordance with present circumstances. But another difficulty is rapidly increasing, through the misguided action of Boards of Guardians, in reference to the supply of nurses for the larger metropolitan and provincial infirmaries. We may take the proceedings at the last week's meeting of the Chelsea Guardians as a text. It is always customary in the case of these large "State hospitals" that the initial selection of nurses shall rest in the hands of the head of the nursing department—the matron—and in the case of the Chelsea Infirmary, whilst this course was followed, no difficulty was experienced by Miss de Pledge in obtaining applications in answer to her advertisements. But about a year since some members of the Board considered it advisable to take this matter of the selection into their own hands, and, in consequence of their action, advertisements were issued from the clerk instead of the matron. The natural result ensued. Competent women will not apply for appointment if they believe their selection rests with an unexpert body, and the inference they naturally draw from such an advertisement is that either the matron is incompetent, and possibly untrained, or that the status of the nurses is not what it should be. For the past year, therefore, the Chelsea Guardians have been practically unable to obtain any suitable replies to their perpetual advertisements, and they are now between the horns of a dilemma. It is much to be regretted that a lady Guardian is one of the chief supporters of this absurd system, but the Chelsea Board fortunately possesses an admirable chairman in Mr. Brass, and that the Board generally is realising the mistake they have made was shown last week, when a recommendation was adopted that "all nurses go before the matron for approval before coming before the Board." The matter is to be further discussed at this week's meeting, when it is to be hoped that the Board will show their good sense by leaving this matter of the selection of probationers and nurses in the proper hands. It is manifestly ridiculous to require a matron to administer her department properly if she is practically allowed no voice in the selection of those who are to work under her direct control. This, however, by the way. The point is that the best nurses will not work under lay management, and that if Guardians wish for good nurses it seems a pity to make regulations and to word advertisements in such a form as to prevent their applying, especially when, in fact, their infirmary is one which in every other respect ought to be attractive.

### Where to Go.

ROYAL BRITISH NURSES' ASSOCIATION.—On Friday, February 24th, at six p.m., Mrs. Garrett Anderson will deliver the third sessional lecture at 11, Chandos Street, Cavendish Square, on "The History and Effects of Vaccination." The chair will be taken by Sir Thomas Smith, Bart., and the lecture will be preceded by a reunion of members, when tea and coffee can be obtained. Admission is limited to members and their friends.

### "Mental Hygiene as a Basis for Character Formation."

"MENTAL Hygiene as a Basis for Character Formation" was the text of Mrs. J. White Wallis's thoughtful address to the Childhood Society on the 21st inst. at the Sanitary Institute, Margaret Street. Mrs. White Wallis is well fitted to speak with authority, inasmuch as both by study and experience she has qualified herself in the art of building up the character of the young by teaching and training. She told her hearers that she had closely studied some 40 or 50 of the pupils that had passed under her observation from infancy to youth, from youth to maturity. She cautioned them against sacrificing one portion of the individual's capacity to another, one period of the lifetime to another. The just balance of qualities, the unfolding of the character, the perfecting of all for the whole life is the true purpose of education. A perfect rose is the right development of the perfect bud, and the deformed, mutilated bud will never grow into a perfect blossom. After giving some practical rules for healthy school buildings, warmth of the room and of the student, position of the body, length of class, times for recreation, all relating to the acquirement and maintenance of physical health, Mrs. White Wallis expressed her opinion that students should not be so over-fatigued that lengthy periodical holidays were necessary, but that the balance of health should be constantly maintained. As soon as a child was tired it should be allowed to drop out of class, and there should be no home lessons. It had been proved that five hours' study a day was half an hour too long. She found that mixed schools were growing in favour by those who had tested them. Girls lost many petty vanities, became braver, more vigorous, and acquired greater scientific precision of thought and expression. Boys became less rough, more sympathetic, tidier, less arrogant in their attitude towards girls. Contrary to expectation, the facility of intercourse checked, rather than encouraged, sentimentality; and the tendency to write love letters was considerably lessened. To awaken original thought, to teach the young to see clearly and truly, to instruct from the concrete to the abstract, to remember that books contain only the records of knowledge and not knowledge itself, to surround the child with an environment of truth and righteousness were all points full of interest, which the lecturer showed lay at the root of right education.

The discussion following the address was unimportant; but Dr. Warner's statement that mental hygiene and education produced a larger brain, and, in consequence, conduced to longevity, was interesting.

### Novelties for Nurses.

### A NEW DRESS LINING.

THE nicest lining we have yet seen is the "Nubian Fast Black Lining." It possesses the qualities of silk, in fact it closely resembles the softest and finest of watered silks. The dye is fast, the width is from 40 to 36 in., and it is durable and cheap. The "Cameron" quality, which is the best, is only 9¾d. the yard, whilst an excellent quality can be had for 5½d. per yard. It has hitherto been impossible to obtain a lining as firm, light, and soft as silk, at a small cost, but the difficulty has been solved in the "Nubian Fast Black" linings, which we strongly recommend to our readers.

### A Book and its Story.

### BISHOP WALSHAM HOW.

The Life of William Walsham How,* first Bishop of Wakefield, has been given to us with commendable promptitude, only some fourteen months after his death, and appears at an opportune moment, when the minds of many are grieved at the agitations raging in the Church. Walsham How was a strong man, but a man of peace. No statesman like a Tait, no brilliant orator like a Magee, no sparkling wit like a "Samuel of Oxford," he was a true active Christian saint, and left a deep impress for good on the spiritual life of the Church. A brief sketch of his career must suffice. Going up to Oxford in 1841, the variety of his reading and pursuits kept him from the narrow extravagances of the extreme Oxford school, and though his degree suffered in consequence, his wide influence in after life amongst all sections of Church people must be traced to this breadth of view and his power of looking at two sides of a question. He served as curate at Kidderminster under its famous "Hero Vicar," and his 28 years as rector of Whittington, a country parish in Salop, proved a fitting prelude to his after career, marked as they were by his tactful dealing with the clergy of his rural deanery (three of whom in those unregenerate days of Church life had not even fonts in their churches), by his careful levelling up of the Church tone in his parish and the neighbourhood, and by that interest in school work and the children which afterwards earned for him the name of "the children's Bishop." In 1879, at the age of 56, he was called from the country, which he so loved, to take the oversight of East London, then "an ecclesiastical Botany Bay," and such was the vigour with which he threw himself into the work as "leader of the East London crusade," that before he left in 1887 he had consolidated all parties, he had drawn out the sympathy of the West End for their poorer brethren in the East End, and though he did not originate the practice of "slumming," he was largely instrumental in establishing the system of University and public schools, settlements which have done so much for the social and moral uplifting of the masses. He loved to tell how when he began to work amongst them the Eastenders would look with amazement at his "shovel and gaiters" and say, "There's *a* Bishop," how it soon changed into "There's *the* Bishop," and was for years before he left, "There's *our* Bishop." After refusing Manchester, he accepted the task of organising the new diocese of Wakefield in his 65th year, and though sorely tried at first by the apathy of Church people in responding to his calls upon them and by the divisions among the clergy and their neglect of duty in some cases, his courage and loving sympathy triumphed over all obstacles, and when, in 1897, he was called to rest, none mourned his loss more than those of his diocese of Wakefield, who had found in him, indeed, a true Father in God. As a Churchman, Bishop How's position was that of the old High Church school—the Anglican as distinct from the Anglo Catholic or Ritualist party. "That wretched Ornaments Rubric," he admitted, might allow of more than he himself approved; he was careful to impress on his clergy the need of frequent Communions, the keeping of festivities and daily services; he condemned evening Communions; he believed in the Real Presence; but, on the other hand, he refused to define the manner of that Presence; he deprecated non-communicating attendance, and looked on fasting Communions only as a means to an end; he loyally accepted the Lincoln judgment as far as it went; and further, he was "not one of those who decry or despise the verdict of civil courts in ecclesiastical matters. The State has its rights as well as the Church"; and on questions such as Confession, Prayers for the Dead, and the like his common sense helped him to the more moderate view that they may be permissible but are not be enjoined. In a letter to a friend he writes: "I entirely agree in dreading the language used by the advanced party as to Holy Communion. It is not faithful to our Church nor to the Bible"; but none the less was he completely out of sympathy with the "Protestant" who closes his church from week end to week end, and provides few and meagre spiritual opportunities for his people. Again he says, "I do earnestly hope that the young men of the present day will not forsake the grand old divines of our own Church, *e.g.*, Jeremy Taylor, Hooker, Jackson, Waterland, &c.,—for the pamphlets and newspaper controversies, which are really the staple of many a man's divinity in these days of haste and shallowness." Great as was his personal influence over those with whom he came in contact at ordinations, missions, and retreats, he reached a far wider circle with his writings, especially his volumes of "Plain Words," his "Commentary on the Four Gospels," and, above all, his "Pastor in Parochia," which nine out of ten of the younger clergy have in constant use. To the general public his fame was even greater as a hymn writer, the popular favourites being "For all the Saints who from their Labours Rest," and "O Jesu, Thou art standing," while his recent Jubilee hymn, "O King of Kings," is still fresh in our memory, especially those lines—

> "God make the world a better world
> For man's brief earthly dwelling:"

a prayer to which he had tried to give expression all his life. The volume, which concludes with some chapters on "the Bishop as a botanist," "the Bishop as a fisherman," and some "Letters on spiritual matters," will be found to give an accurate portrait of his life in all its phases, but like so many of such works, it lacks consistency and evenness. In the earlier half extracts and letters are judiciously worked up into a consecutive whole; but the more important periods of the Bishop's life, in East London and at Wakefield, are treated less happily and less consecutively than they deserve. On the whole we get a clear insight into the life of the man, but many will regret that the effect of his work on the Church at large, and especially in London, is not more clearly brought out. But it must be remembered that Bishop Walsham How was essentially a man who left his mark on the inner life, rather than on the external status, of the Church. Childlike in simplicity, untiring in energy, endowed with a keen sense of humour, intensely sympathetic, and consistent from his earliest years in holiness, his Churchmanship was marked by that plain commonsense so often lacking in the holiest of men, and therein lay the secret of his influence. He was no great social reformer, nor did he take an active part in legislating for the amelioration of the material condition of the masses amongst whom he laboured, though his work compelled the attention of others to it. He laboured always for the spiritual good of his fellow-creatures; and with the knowledge of this the writer of the memoir has purposely perhaps portrayed the more lasting side of his father's work, his influence for good over others. It may may be better so: the life may thus be more correctly drawn, but the book undoubtedly loses in public interest by reason of this treatment.

* "Bishop Walsham How. A Memoir." By his Son (Frederick Douglas How). (London: Isbister and Co. 1898. Price 16s. Demy 8vo.)

### Pension Fund Nurses.

### MISS BURNS' WEDDING GIFT.

We have still further contributions to acknowledge, viz: Nurses M. E. Smith and C. Dunn, Policy No. 36 (Sydney).

### Everybody's Opinion.

[Correspondence on all subjects is invited, but we cannot in any way be responsible for the opinions expressed by our correspondents. No communication can be entertained if the name and address of the correspondent is not given, as a guarantee of good faith but not necessarily for publication, or unless one side of the paper only is written on.]

### AFFLUENT BREADWINNERS.

"AN EX-HOSPITAL SISTER" writes: I am extremely glad to see the letter of "Another Who Loves Nursing" in this week's issue. My opinion is that those who take up nursing simply as a means of livelihood, without having any real love for the work, never make what I call good nurses. They may learn their profession and be mechanical helps to the doctor. That a good machine is very useful at times I do not deny, but they are not nurses in the highest sense of the word, and never will be unless they have within them the love to their fellow creatures and the greatest thing in the world, "the love that suffers long and is kind." Everyone is not born a nurse. So few have the sympathetic feeling for their patients which enables them to do and say the right thing at the right time. Those who cannot do this let them take up any other profession than nursing.

### NO LONGER ABLE TO WORK.

"NURSE ADA" writes: In reply to "Sympathetic," I do not consider monthly training alone at all suitable for nursery nurses; you want to be trained beyond a month's care of infants to take up that work. I should advise nurses to train at the Norland Institute, or if they cannot afford that to go under a good head nurse and gain their experience. I certainly agree with "Sympathetic" that there is a good opening for nursery nurses, and the pay is much better—as I was trained three years ago at Queen Charlotte's; previously I had been lady nurse, and have since had monthly cases, and found it too hard work to continue. Now I have charge of a delicate infant, and am getting a salary of £48 all found; my sister, who is a thorough trained nurse of ten years, can only get £36. I should advise all young nurses to train as thorough nursery nurses. It may not be such a bright life as that in a hospital, for we all know it is very monotonous to be always with children, but as one learns to love them that soon wears off.

### Appointments.

THE NATIONAL SANATORIUM FOR CONSUMPTION AND DISEASES OF THE CHEST, BOURNEMOUTH.—Miss Helen Todd was appointed Lady Superintendent and Matron of the above sanatorium on February 6 h. She was trained at St. Bartholomew's Hospital, London, and passed the L.O.S. examination from the Lying-in Institution, West Street, Brighton, where also she acted as locum tenens both for the superintendent of midwifery pupils and for the matron. Since May, 1898, Miss Todd has been assistant matron of the Brentwood Poor Law Schools, having had charge of the infirmary there.

BOROUGH HOSPITAL, WOLVERHAMPTON.—On January 27th Miss E. J. Mulligan was appointed Matron here. She received three years' training at the Sir Patrick Dun's Hospital, Dublin, where she subsequently became sister of the female surgical and obstetric wards, and night superintendent. For three years Miss Mulligan was sister of the constabulary wards at Dr. Steevens' Hospital, Dublin, and for the last eleven years she has been matron of the Borough Hospital, Kidderminster.

VICTORIA NURSES' HOME, GODALMING.—Miss Elizabeth von Stieglitz has been appointed Lady Superintendent of the above home. She was trained at Addenbrooke's Hospital, Cambridge, and afterwards became nurse at the New Hospital for Women, Euston Road, London; and sister of the female accident and surgical ward, Sussex County Hospital, Brighton. Miss von Stieglitz obtained her L.O.S. certificate after training at the Clapham Maternity Home.

### Examination Questions for Nurses.

"SISTER SOPHIA" is the winner of the first prize for this month's question, and "Nurse H. Bell" takes the second. It is pleasant to see how many competitors we have, showing that the interest in these practical matters remains undiminished. Now for a word of friendly advice. "Whatever is worth doing at all is worth doing well" is an invaluable adage, but one too often forgotten. A large proportion of the papers submitted are so badly written as to be unreadable, and so slovenly and careless that one cannot but feel that the writer must also be slovenly in her work. With regard to the answers themselves, there are many showing thought and practical powers of contrivance, but the larger number make the mistake of raising the patient's feet or giving him something to push against; the former is useless in a sitting position, and is naturally not needed in a recumbent one, and the latter, though useful as an auxiliary means, will not obviate the invariable tendency to sink heavily in the bed, because the weight of the body causes it to gravitate surely downwards. This, then, must be the point of attack; make a counter traction, as explained by Sister Sophia, and the result will be success.

### FIRST PRIZE.

A simple plan to prevent a helpless patient slipping down in bed is to take a bolster, tie at each end with a piece of bandage, place the middle of the bolster under the patient's thighs and tie the bandages to the head of the bed. I have seen that succeed when elaborate arrangements failed. Any clean long bag could take the place of the bolster. Failing a better material, it might be filled with torn strips of paper. Any strong string would secure it, and, failing a head to the bed, two nails might be driven into the wall.—SISTER SOPHIA.

### SECOND PRIZE.

Two methods may be used. (1) Give the patient something firm to press his feet against, such as a hassock or a block of wood covered with a shawl or any soft material. (2) Where the first method is not practicable, owing to the absence of a footboard to the bed, another plan may be adopted. Take a small pillow, cushion, or rug, and roll it into the form of a bolster and fasten it so with safety pins or tapes; then through the centre draw a long, narrow strip of calico or a strong bandage, about five or six yards long, and having placed the bolster under the patient's thighs, tie the ends of the bandage, one on either side, to the top of the bed; but not too high up, else it will interfere with the patient's elbows. Another pillow may be placed under the knees.—H. J. B.

Answers to questions, which may be signed with a nom-de-plume, but accompanied by the sender's name and address, will be submitted for expert opinion, and the replies to the questions considered to be the best will be published, and the writer will be offered the choice of a useful book from the catalogue of the Scientific Press. Answers, marked "Examination Questions," must reach the Editor, 28, Southampton Street, Strand, within one fortnight of the publication of the paper in which the question appears.

### QUESTION FOR MARCH.

Give your ideas of the best plan for supporting a patient with the least possible fatigue whilst undergoing the water cure; that is to say, when the treatment consists of immersion in a bath for many hours at a time.

Answers must not exceed 600 words.

### Wants and Workers.

MISS E. E. BOYCE, Mackenzie Street, Slough, and Mr. H. F. Willeringhaus, 51 and 52, Aldermanbury, E.C., will be pleased to send their copies of THE HOSPITAL free to any nurse or institution which cannot afford to subscribe for it.

